# The CB1R of mPFC is involved in anxiety-like behavior induced by 0.8/2.65 GHz dual-frequency electromagnetic radiation

**DOI:** 10.3389/fnmol.2025.1534324

**Published:** 2025-03-12

**Authors:** Bin Sun, Teng Xue, An-ning Gao, Xin-yu Wang, Shuang Wu, Xiao-man Liu, Li-hui Zhang, Meng-hua Li, Dong-fang Zou, Yan Gao, Chang-zhen Wang

**Affiliations:** ^1^Beijing Institute of Radiation Medicine, Beijing, China; ^2^Department of Neuroscience, School of Life Sciences, Southern University of Science and Technology, Shenzhen, China; ^3^Center of Cognition and Brain Science, Beijing Institute of Basic Medical Sciences, Beijing, China

**Keywords:** anxiety-like behavior, endocannabinoid system, medial prefrontal cortex, amygdaloid nucleus, cannabinoid receptor type 1

## Abstract

As mobile phones and communication base stations become more widespread, concerns have arisen regarding the potential risks of environmental exposure to multi-frequency electromagnetic radiation (EMR) and its effects on mental health. To address these concerns, our study established a dual-frequency EMR mouse model at 0.8/2.65 GHz to explore potential molecular mechanisms and intervention targets. Our results revealed that exposure to this dual-frequency EMR significantly induced anxiety-like behavior in mice. Molecular experiments further showed a significant decrease in cannabinoid receptor type 1 (CB1R) levels in the medial prefrontal cortex (mPFC) of the mice, along with a notable reduction in the endogenous cannabinoids 2-arachidonoylglycerol and anandamide. This led to a downregulation of the entire endocannabinoid system (ECS). Additional confirmation was obtained by overexpressing and knocking down CB1R in the mPFC. We found that increasing mPFC CB1R levels could effectively reduce anxiety-like behavior, while decreasing mPFC CB1R levels exacerbated it. Furthermore, we found dual-frequency EMR induced the change of ECS in the basolateral amygdala (BLA). Notably, female mice exhibited similar behavioral phenotypes and molecular mechanisms in response to dual-frequency EMR. In summary, our study demonstrates that anxiety induced by dual-frequency EMR is closely linked to the function of the ECS in the mPFC and BLA, and that CB1R expression in the mPFC plays a significant role in modulating emotional behavior in mice.

## 1 Introduction

Electromagnetic radiation (EMR) refers to the phenomenon of electromagnetic energy radiating into space in the form of electromagnetic waves (Rohrlich, 1961). With technological advancements, people are inevitably affected by EMR from mobile phones and other wireless communication devices, potentially resulting in symptoms such as nervous exhaustion, fatigue, headaches, sleep disturbances, reduced attention, anxiety, and depression (Glaser and Brown, 1976; Raines, 1981). Electromagnetic waves in the 0.8 and 2.65 GHz frequency bands, which are important for mobile phone usage (Zheng et al., 2023), have raised significant concerns regarding their impact on human health.

The mechanism by which complex-frequency EMR induces negative emotions remains unclear. The endocannabinoid system (ECS) plays a critical role in regulating anxiety and depressive emotions (Lu and Mackie, 2015; Lutz et al., 2015). Postsynaptic excitation triggers the release of the endocannabinoids 2-arachidonoylglycerol (2-AG) and anandamide (AEA), which, through retrograde signaling, activate presynaptic CB1R to inhibit neurotransmitter release at the presynaptic terminal, a process involved in emotional regulation (Zou and Kumar, 2018). In previous studies, our group found that dual-frequency EMR-induced anxiety-like behavior in mice is associated with the endocannabinoid system (Xue et al., 2024), although the specific brain regions involved remain unclear. The medial prefrontal cortex (mPFC) is involved in the modulation of emotional responses, and recent studies have demonstrated that the activation of endocannabinoid signaling within this region can reduce anxiety-like behaviors. The mPFC's ECS, particularly through CB1 receptors, has a significant impact on emotional regulation by influencing neural circuits that govern fear and anxiety responses (Imperatore et al., 2015). Furthermore, inhibition of endocannabinoid system function in the mPFC led to an increase in anxiety-like behavior in mice, further highlighting the critical role of the ECS in the regulation of anxiety (Demaili et al., 2024). Therefore, The ECS of mPFC plays a crucial role in emotion regulation. However, it remains unclear whether the ECS of mPFC is involved in dual-frequency EMR-induced anxiety.

The endocannabinoids (eCBs) are dynamically signal in mPFC and BLA neurons and contribute to adverse mood extinction. It suggests that eCBs in the mPFC and BLA are potential therapeutic targets for extinction deficit disorders, including anxiety and PTSD (Gunduz-Cinar et al., 2023). Furthermore, after stress, activation of the BLA-mPFC pathway occurs, and 2-AG-mediated endocannabinoid signaling can inhibit synaptic glutamate release in the BLA-mPFC circuit, thereby alleviating anxiety (Marcus et al., 2020). Research has shown that activation of the ECS in the BLA can reduce anxiety, the activation of CB1R in the BLA reduces anxiety-like behavior in rodents (Morena et al., 2016). Inhibiting the breakdown of endocannabinoids in the BLA led to a decrease in anxiety-like behaviors in mice (Kondev et al., 2023). These findings indicate that the ECS in the BLA regions is also involved in the regulation of emotions.

However, it remains unclear whether dual-frequency EMR induces changes in the ECS in brain regions closely associated with emotional regulation, such as the medial prefrontal cortex (mPFC) and basolateral amygdala (BLA), including variations in CB1 receptors (CB1R), 2-AG, AEA, and enzyme levels. This study aims to elucidate the specific brain regions, molecular mechanisms, and intervention targets that contribute to anxiety in mice through the establishment of a dual-frequency electromagnetic radiation animal model.

## 2 Results

### 2.1 Induction of anxiety-like behavior in male mice exposed to dual-frequency (0.8/2.65 GHz) electromagnetic radiation

**Figure 1:**
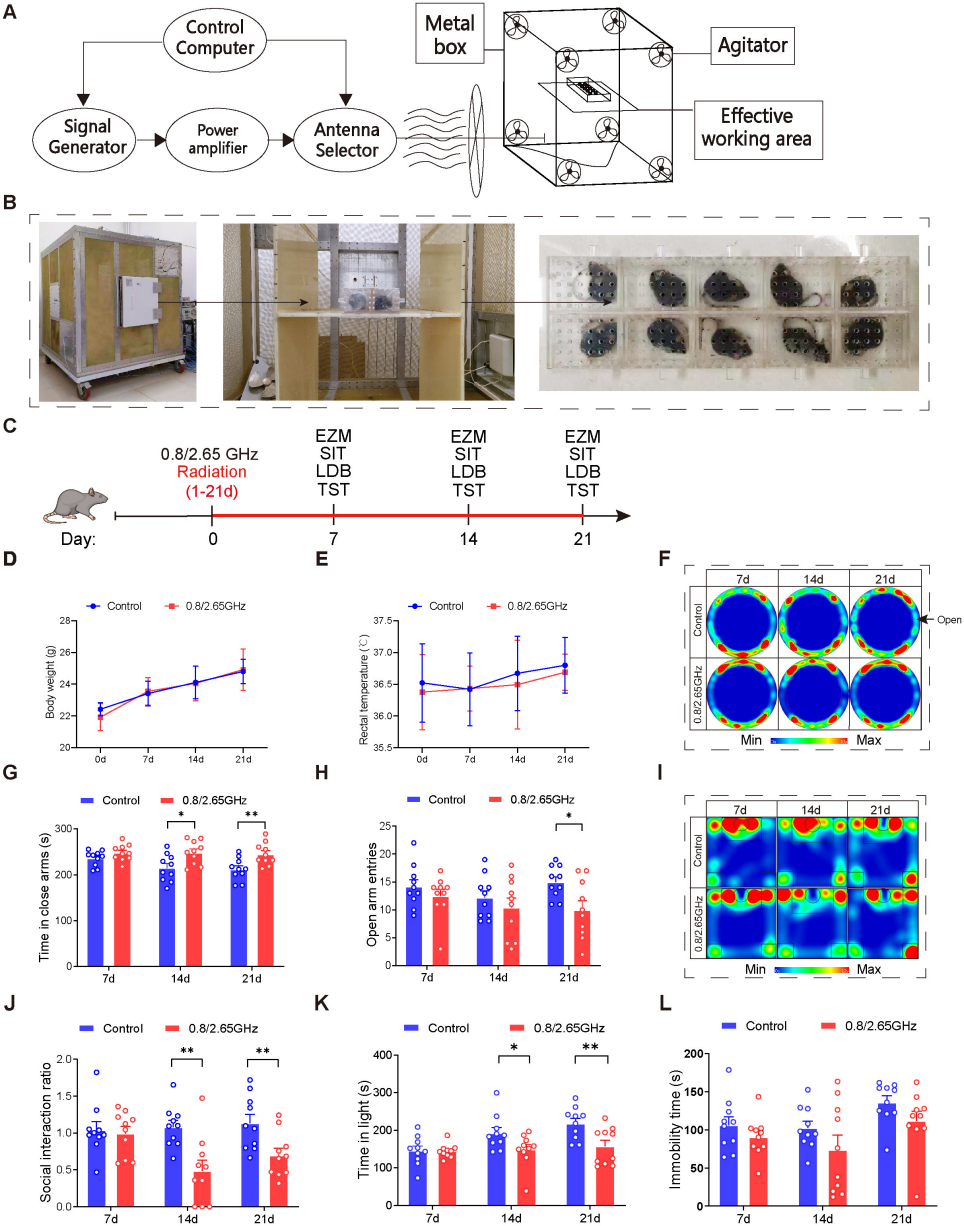
Effects of 0.8/2.65 GHz dual-frequency electromagnetic radiation on the emotional behavior of male mice. Schematic diagram **(A)** and actual image **(B)** of the working principle of the electromagnetic reverberation chamber. **(C)** Behavioral timeline of dual-frequency electromagnetic radiation (0.8/2.65 GHz, 4 W/kg, 4 h/day, total radiation duration of 21 days). **(D, E)** Effects of dual-frequency EMR on body weight and rectal temperature in mice (*n* = 10/group). **(F–H)** Elevated zero maze test (EZM, 7, 14, 21 days, 9:00–11:00); representative statistical heatmaps of EZM results (7, 14, 21 days, *n* = 10/group) **(F)**, time spent in closed arms **(G)**, and number of entries into open arms **(H)**. **(I, J)** Social interaction test (SIT, 7, 14, 21 days, 19:00–21:00, *n* = 10/group); representative statistical heatmaps of SIT results (7, 14, 21 days, *n* = 10/group) (I), and social interaction ratio (SIR) **(J)**. **(K)** Light-dark box test (LDBT, 7, 14, 21 days, 20:00–22:00, *n* = 10/group), time spent in the light box **(K)**. **(L)** Tail suspension test (TST, 7, 14, 21 days, 9:00–11:00, *n* = 10/group), relative immobility time. All data are expressed as means ± SEM, *p < 0.05, **p < 0.01, control vs. 0.8/2.65 GHz, repeated-measures analysis of variance in **(B, C)**. Unpaired *t*-test in **(G, H, J, K, L)**.

The EMR devices used in this study are shown in Figures 1A, B. Mice in the radiation group were placed in the effective working area for exposure, while mice in the control group were placed in the same area but with the devices turned off. Mice were randomly assigned to control and dual-frequency (0.8/2.65 GHz) EMR groups. The dual-frequency group underwent exposure to 0.8 GHz radiation for 2 h, followed by 2.65 GHz radiation for an additional 2 h, totaling 4 h (8:00 AM−12:00 PM) per day, with a specific absorption ratio (SAR) of 4 W/kg, excluding the days designated for behavioral testing (e.g., days 7, 14, and 21).

Mouse body weight and rectal temperature were measured during radiation exposure, and no differences were found between the control and dual-frequency EMR groups (Figures 1D, E). After radiation exposure, we adopted behavioral paradigms for evaluation (Figure 1C). In the elevated zero maze (EZM) test (Figures 1F–H), the dual-frequency EMR group showed a significant increase in the time spent in the closed arms on days 14 and 21 (Figure 1G, *p* = 0.0139, day 14; *p* = 0.0071, day 21), along with a significant decrease in entries into the open arm on day 21 (*p* = 0.0157, Figure 1H) compared to the control group. In the Social Interaction Test (SIT) test (Figures 1I–J), the dual-frequency EMR group exhibited a significant reduction in the social interaction ratio (Figure 1J, *p* = 0.0026, day 14; *p* = 0.0098, day 21) on days 14 and 21 compared to the control group. In the light-dark box (LDB) test, the dual-frequency EMR group spent significantly less time in the light compartment on days 14 and 21 (Figure 1K, *p* = 0.0424, day 14; *p* = 0.0086, day 21) compared to the control group. These findings indicate that dual-frequency (0.8/2.65 GHz) EMR induces anxiety-like behavior in mice. However, in contrast to the control group, the dual-frequency EMR group showed no significant difference in total immobility time during the tail suspension test (TST) on days 7, 14, and 21 (Figure 1L), suggesting that dual-frequency (0.8/2.65 GHz) EMR did not induce depression-like behavior in mice.

### 2.2 Dual-frequency (0.8/2.65 GHz) electromagnetic radiation significantly reduced the expression of *Cnr1* and the content of CB1R in mice medial prefrontal cortex

**Figure 2:**
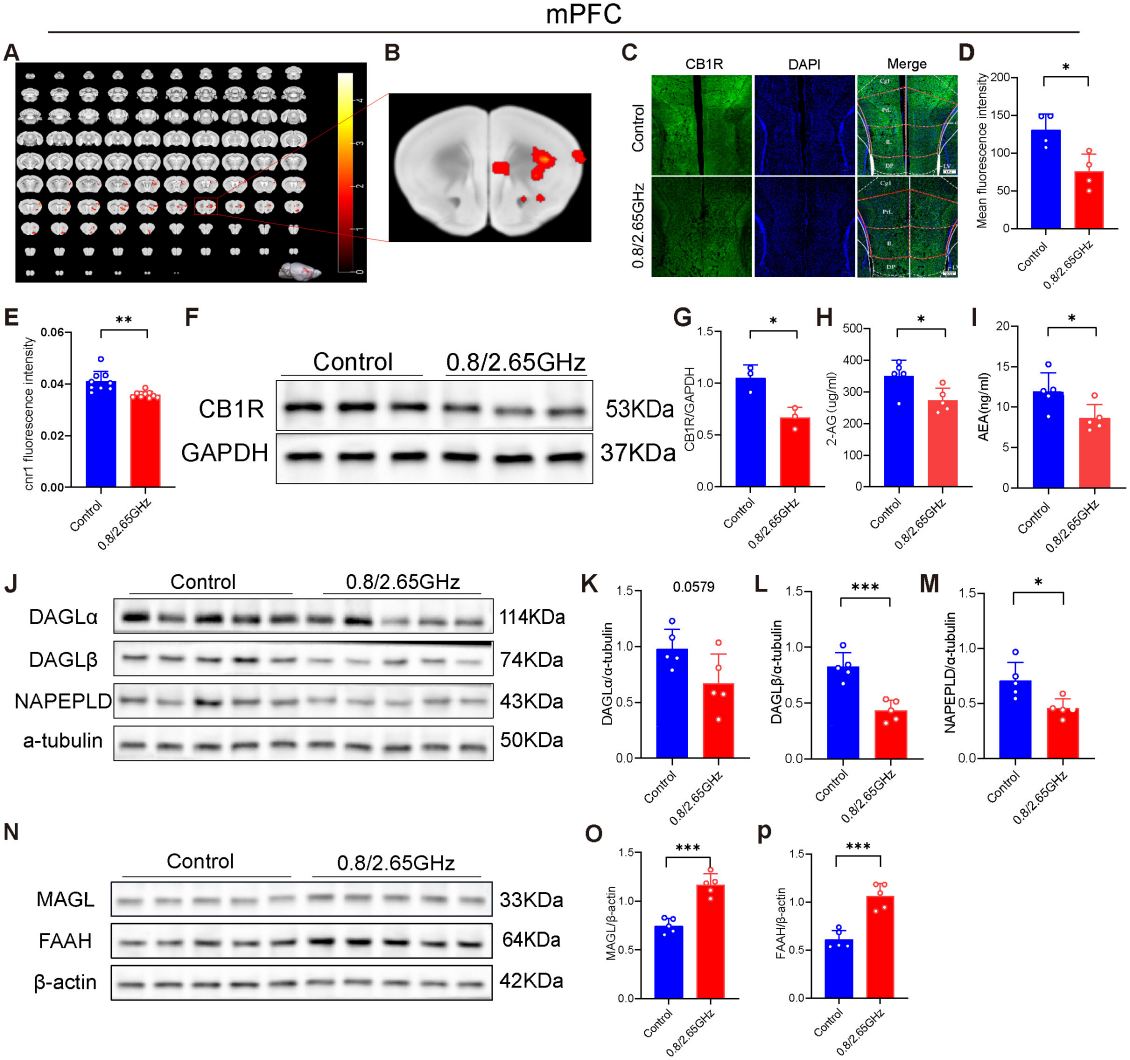
Effects of 0.8/2.65 GHz dual-frequency electromagnetic radiation on the endocannabinoid system in the mPFC of male mice. **(A, B)** Functional magnetic resonance imaging (fMRI) detection of the effects of dual-frequency EMR on brain activity in mice, showing whole-brain scans and a schematic diagram of the mPFC (*n* = 12/group). **(C)** Representative immunofluorescence images showing CB1R, DAPI, and merged images. **(D)** Average fluorescence intensity of CB1R in the mPFC (*n* = 4/group). **(E)** q-PCR analysis of cnr1/β-actin gene expression in the mPFC (*n* = 9/group). **(F)** Western blot images showing target protein CB1R and internal control GAPDH in mPFC. **(G)** Statistical analysis of CB1R/GAPDH Western blot results in the mPFC (*n* = 3/group). **(H, I)** ELISA analysis of 2-AG and AEA molecules in the mPFC (*n* = 5/group). Effects of EMR on cannabinoid synthesis enzymes in the mPFC: **(J)** Western blot results for target proteins (DAGLα, DAGLβ, NAPEPLD) and internal control α-tubulin in the mPFC, and **(K–M)** statistical analysis of Western blot results for (DAGLα, DAGLβ, NAPEPLD)/α-tubulin in the mPFC (*n* = 5/group). **(N–P)** Effects of EMR on cannabinoid hydrolase in the mPFC. Western blot results of target proteins (MAGL, FAAH) and internal control β-actin in the mPFC **(N)**, and statistical analysis of (MAGL, FAAH)/β-actin Western blot results in the mPFC **(O, P)** (*n* = 5/group). All data are expressed as means ± SEM, *p < 0.05, **p < 0.01, ***p < 0.001. All results were analyzed using the unpaired *t*-test.

To further explore which specific brain regions are responsible for inducing anxiety in the mice, we conducted relevant research. First, using functional magnetic resonance imaging (fMRI) technology, we investigated the changes in brain region activity induced by dual-frequency EMR. We found that, compared to the control group mice, the irradiated group mice exhibited a significant increase in the amplitude of low-frequency fluctuations (ALFF) of the medial prefrontal cortex (mPFC), indicating higher activity levels in this brain region (*p* < 0.05, Figures 2A, B); there were also some brain nuclei exhibited a significant increase that were not closely associated with anxiety (*p* < 0.05, Figure 2A), these brain nuclei include AOM (anterior olfactory nucleus, medial part) (Wolf et al., 2024), CPu (caudate-putamen-nucleus) (Wu et al., 2024), Acb (accumbens nucleus) (Li, 2024), S1 (primary somatosensory cortex) (Yue et al., 2025). Subsequently, through immunofluorescence experiments, we found that the CB1R signal was significantly weakened in the dual-frequency radiation group compared to the control group (*p* = 0.0134, Figures 2C, D). We also performed quantitative polymerase chain reaction (Pcr) and Western blotting (WB) detection of mPFC brain tissue, found that in the mPFC, compared to the control group, the *Cnr1* gene encoding CB1R was notably downregulated in the radiation group (*p* = 0.0017, Figure 2E). WB confirmed a significant reduction in CB1R protein expression in the mPFC (*p* = 0.0142, Figures 2F, G), indicating that prolonged exposure to dual-frequency EMR impacted the CB1R expression of the ECS within the mPFC.

Pertaining to the endocannabinoids 2-Arachidonoylglycerol (2-AG) and Anandamide (AEA), crucial ligands of the ECS that predominantly bind to CB1R and regulate mood, we delved into their content. Our analysis revealed a significant decrease in the 2-AG (*p* = 0.0279, Figure 2H) and AEA (*p* = 0.0319, Figure 2I) content in mice mPFC exposed to dual-frequency EMR compared to the control group.

Furthermore, we investigated the enzymes responsible for controlling endocannabinoid levels in the brain. The Western Blot experiment was performed and pictures of endocannabinoid synthase (Figures 2J–M) and endocannabinoid resolvase (Figures 2N–P) were taken. Our investigations found that Diacylglycerol lipase-α (DAGLα) that Synthesizing 2-AG also have a lower tendency (*p* = 0.0579, Figure 2K), and Diacylglycerol lipase-β (DAGLβ) that Synthesizing 2-AG significantly decrease (*p* = 0.0005, Figure 2L), and Monoacylglycerol lipase (MAGL) degrading 2-AG has significant increase (*p* = 0.0001, Figure 2O). N-acylphosphatidylethanolamine phospholipase D (NAPE-PLD) that Synthesizing AEA significantly decrease (*p* = 0.0154, Figure 2M), and Fatty acid amide hydrolase (FAAH) degrading AEA has significant increase (*p* = 0.0002, Figure 2P), suggesting that 2-AG and AEA downregulation of content also due to insufficient synthesis and Excessive degradation.

In summary, these results suggest that the downregulation of CB1R, along with the downregulation of the ligands 2-AG and AEA in the mPFC, collectively leads to the weakening of their binding, resulting in the downregulation of ECS function, ultimately leading to anxiety-like behavior in mice.

### 2.3 The basolateral amygdala of mice was also involved in anxiety induced by dual-frequency electromagnetic radiation

**Figure 3:**
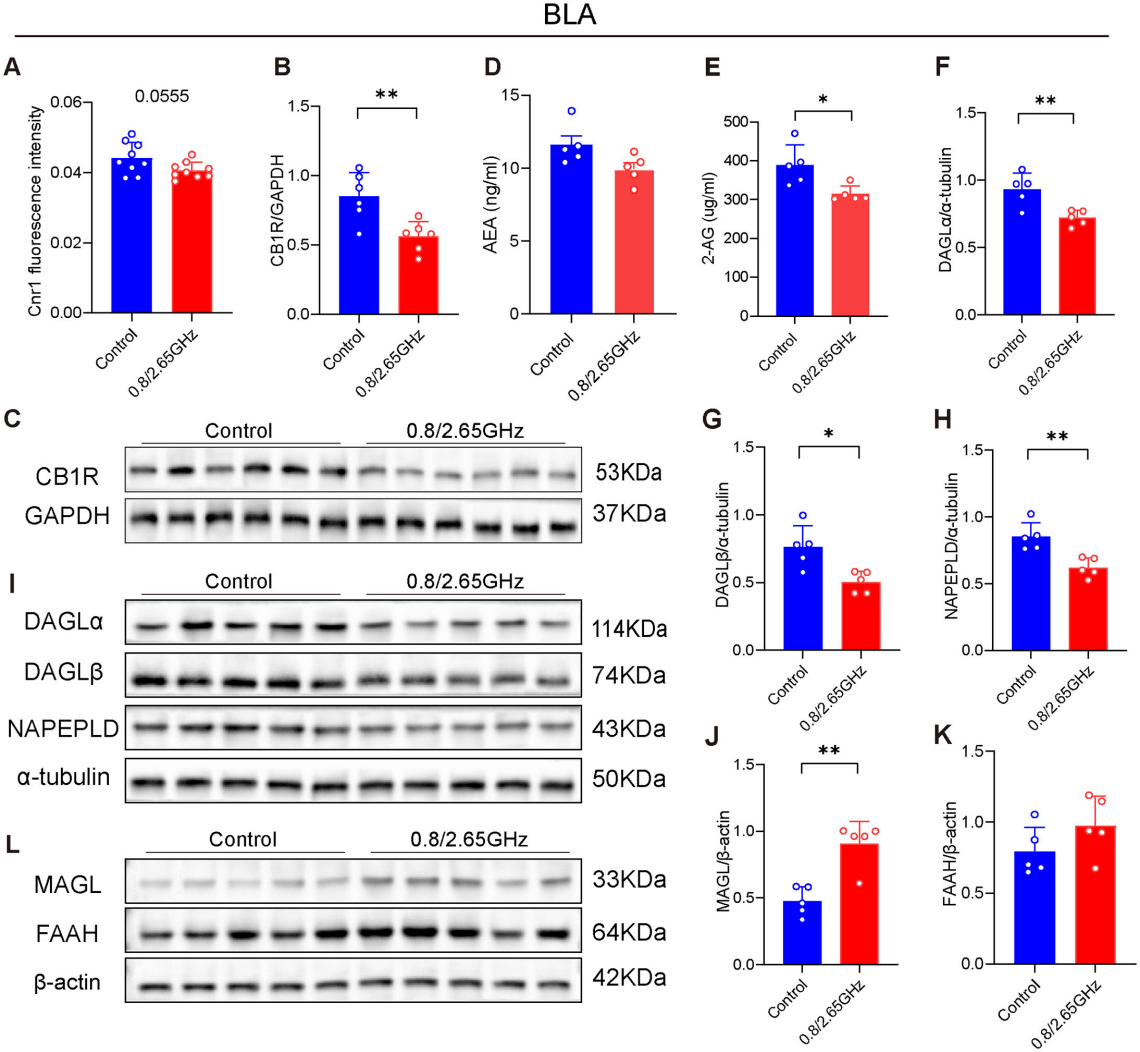
Effects of 0.8/2.65 GHz dual-frequency electromagnetic radiation on the endocannabinoid system in the basolateral amygdala (BLA) of male mice. **(A)** Q-PCR analysis of cnr1/β-actin in the BLA (*n* = 9/group). Changes in CB1R in the BLA, **(B)** statistical analysis of CB1R/GAPDH protein electrophoresis in the BLA (*n* = 5/group), **(C)** schematic diagram of WB results of CB1R/GAPDH in the BLA. **(D, E)** Elisa analysis of 2-AG and AEA molecules in the BLA (*n* = 5/group). **(F–I)** Effects of dual-frequency EMR on cannabinoid synthetase in the BLA, **(I)** WB results of target proteins (DAGLα, DAGLβ, NAPEPLD) and internal reference α-tubulin in the BLA, **(F–H)** analysis of DAGLα, DAGLβ, NAPEPLD/α-tubulin protein electrophoresis in the BLA (*n* = 5/group). **(L–K)** Effects of EMR on cannabinoid hydrolase in the BLA, **(L)** WB results of target proteins (MAGL, FAAH) and internal reference β-actin in the BLA, **(J, K)** analysis of MAGL, FAAH/β-actin WB results in the BLA (*n* = 5/group). All data are expressed as means ± SEM, *p < 0.05, **p < 0.01, All results were analyzed using the unpaired *t*-test.

The base lateral amygdala (BLA) is a downstream nucleus of the mPFC and an important brain region for the regulation of anxiety, and has long been a key target of concern (Feng et al., 2024). Therefore, we have also conducted molecular analysis of the BLA. First, we performed QPcr and WB detection of BLA brain tissue, found that compared to the control group, the *Cnr1* gene encoding CB1R had a lower tendency in the radiation group (*p* = 0.0555, Figure 3A). WB confirmed a significant reduction in CB1R of BLA (*p* = 0.0063, Figures 3B, C), indicating that prolonged exposure to dual-frequency EMR impacted the CB1R expression of the ECS within the mice's BLA.

Similarly, we also examined AEA and 2-AG in the BLA (Figures 3D, E), finding that a significant reduction in the 2-AG content in the BLA exposed to dual-frequency EMR compared to the control group (*p* = 0.0182, Figure 3E). Furthermore, we also investigated their enzymes (Figures 3F–L). We found that DAGLα and DAGLβ that Synthesizing 2-AG significantly decrease (*p* = 0.0075, Figure 3F; *p* = 0.0103; Figure 3G), and MAGL degrading 2-AG has a significant increase (*p* = 0.0013, Figure 3J), suggesting that 2-AG downregulation of content is due to in decrease its synthesizing enzyme and an increase in its hydrolyzing enzyme. Similarly, NAPE-PLD, the enzyme responsible for synthesizing AEA, showed a significant decrease (*p* = 0.0038, Figure 3H). However, FAAH, the enzyme that degrades AEA, showed no significant change, which may explain why the AEA content did not significantly change.

In summary, these results suggest that the downregulation of CB1R within the ECS in the BLA, along with the reduction of 2-AG, may reduce their binding each other, impair ECS function, and ultimately lead to anxiety-like behavior in mice.

### 2.4 Overexpression of CB1R in the mPFC improves anxiety-like behavior induced by dual-frequency electromagnetic radiation

**Figure 4:**
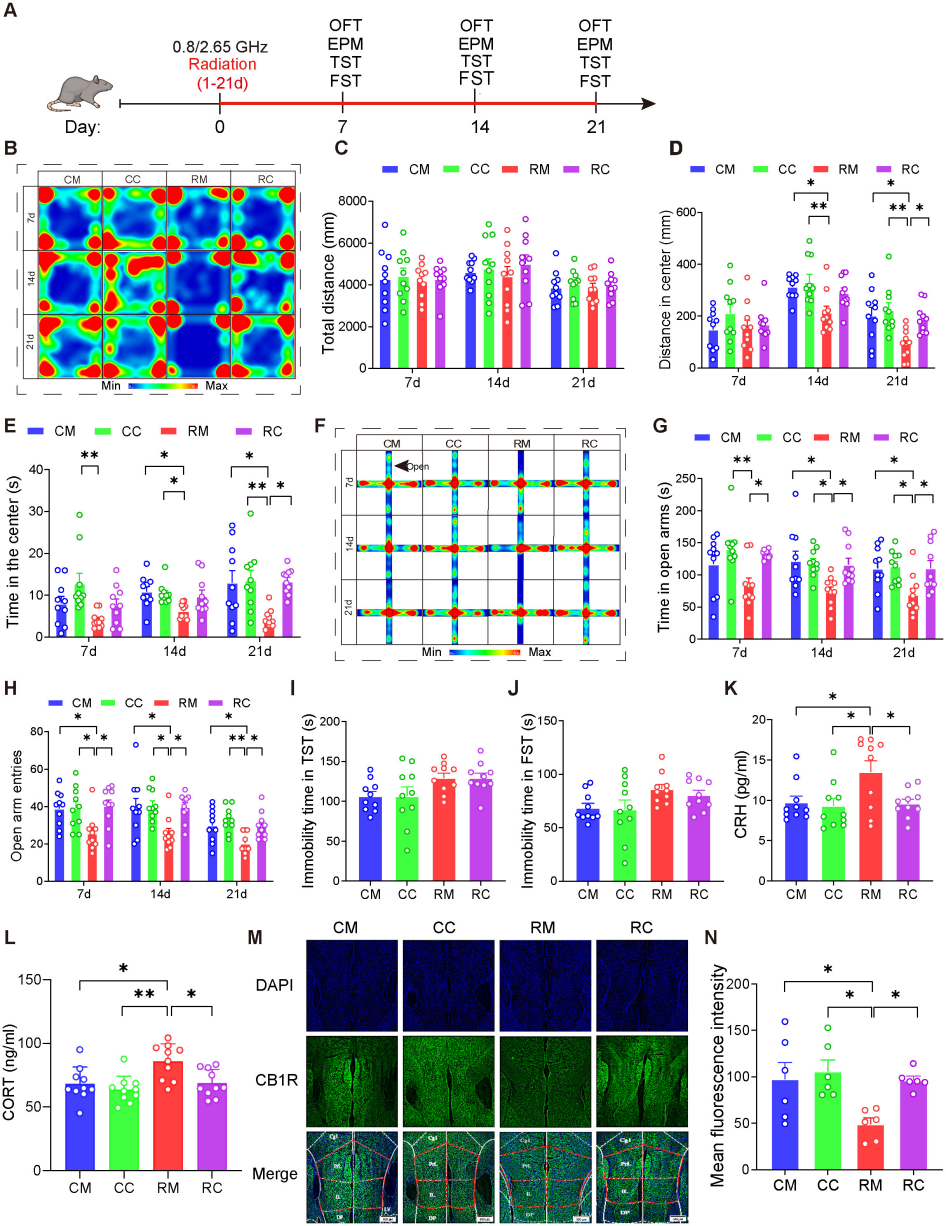
Overexpression of CB1R in the mPFC significantly alleviated anxiety in male mice induced by dual-frequency electromagnetic radiation. **(A)** Time axis of dual-frequency electromagnetic radiation (0.8/2.65 GHz, 4 W/Kg, 4h/day, 21 days) (RM group: 10:00–12:00, 0.8 GHz; 12:00–14:00, 2.65 GHz; RC group: 14:00–16:00, 0.8 GHz; 16:00-18:00, 2.65 GHz). **(B–E)** Open field test (OFT, 7d, 14d, 21d, 8:00–12:00); Representative heat map for statistical analysis of OFT results (*n* = 10/group) **(B)**, total distance **(C)**, central distance **(D)**, and time spent in the center **(E)**. **(F–H)** Elevated plus maze test (EPM, 7d, 14d, 21d, 18:00–22:00); Representative heat map for statistical analysis of EPM results (*n* = 10/group) **(F)**, time spent in the open arms **(G)**, and the number of open arm entries **(H)**. **(I)** Tail suspension test (TST, 20d, 18:00–22:00), relative immobility time (*n* = 10/group). **(J)** Forced swim test (FST, 22d, 8:00–12:00), relative immobility time (*n* = 10/group). **(K)** Serum CRH levels as determined by ELISA (*n* = 10/group). **(L)** Serum CORT levels as determined by ELISA (*n* = 10/group). **(M, N)** Immunofluorescence of CB1R in the mPFC: Representative CB1R, DAPI, and merged images **(M)**, and mean CB1R fluorescence intensity in the mPFC (*n* = 6/group) **(N)**. All data are expressed as means ± SEM, *p < 0.05, **p < 0.01. All results were analyzed using the one-way ANOVA. CM, Control + MCS; CC, Control +Cnr1; RM, Radiation (0.8/2.65 GHz) + MCS; RC, Radiation (0.8/2.65 GHz) + Cnr1.

Given the pivotal role of the ECS in emotion regulation and the observed changes in ECS activity in the mPFC of mice exposed to dual-frequency EMR in this study, our next objective was to investigate whether increasing ECS activity in the mPFC could mitigate neurobehavioral harm. After 21 days of injecting either Cnr1 (pcAAV-CMV-EGFP-P2A-Cnr1-3xFLAG-WPRE; 4.45E+12 v.g./ml) or MCS (pcAAV-CMV-EGFP-P2A-MCS-3xFLAG-WPRE; 3.57E+13 v.g./ml, diluted 8 times to 4.46E + 12 v.g./ml) into the mPFC, we conducted EMR modeling and behavioral tests (Figure 4A).

The results showed that (Figures 4B–H), in the OFT, compared with the RM (Radiation 0.8/2.65 GHz + MCS) group, the RC(Radiation 0.8/2.65 GHz + Cnr1 Overexpression) group displayed increased center distance (*p* = 0.0421, Figure 4D) and time (*p* = 0.0126, Figure 4E) on day 21. In the EPM test, the RC group showed a significant increase in both open arm time (*p* = 0.0247, day7; *p* = 0.0325, day 14; *p* = 0.0355, day 21) and entries (*p* = 0.0137, day 7; *p* = 0.0221, day 14; *p* = 0.0275, day 21) compared to the RM group across all time points (Figures 4G, H). But there is no difference between RM and RC group for tail suspension test (TST) (Figure 4I) and Forced Swimming Test (FST) (Figure 4J). These results demonstrate that overexpression of CB1R in the mPFC significantly alleviated their anxiety-like behaviors induced by dual-frequency EMR.

Moreover, we assessed serum hormone levels and found that the RC group had significantly lower CRH (*p* = 0.0348, Figure 4K) and CORT (*p* = 0.0178, Figure 4L) compared to the RM group, suggesting that overexpression of CB1R in the mPFC restores CORT and CRH levels in response to dual-frequency EMR exposure. Additionally, CB1R expression was significantly higher in the RC group (*p* = 0.0434, Figures 4M, N) compared to the RM group, with no significant difference between the RC and (Control + MCS) CM groups. These findings indicate that dual-frequency EMR downregulates CB1R in the mPFC, and its overexpression can counteract this effect.

In conclusion, overexpression of CB1R in the mPFC may ultimately improve anxiety-like behavior induced by dual-frequency EMR by restoring HPA axis serum hormone levels and CB1R content in the mPFC.

### 2.5 Knock-down of CB1R in the mPFC aggravated anxiety-like behavior induced by dual-frequency electromagnetic radiation

**Figure 5:**
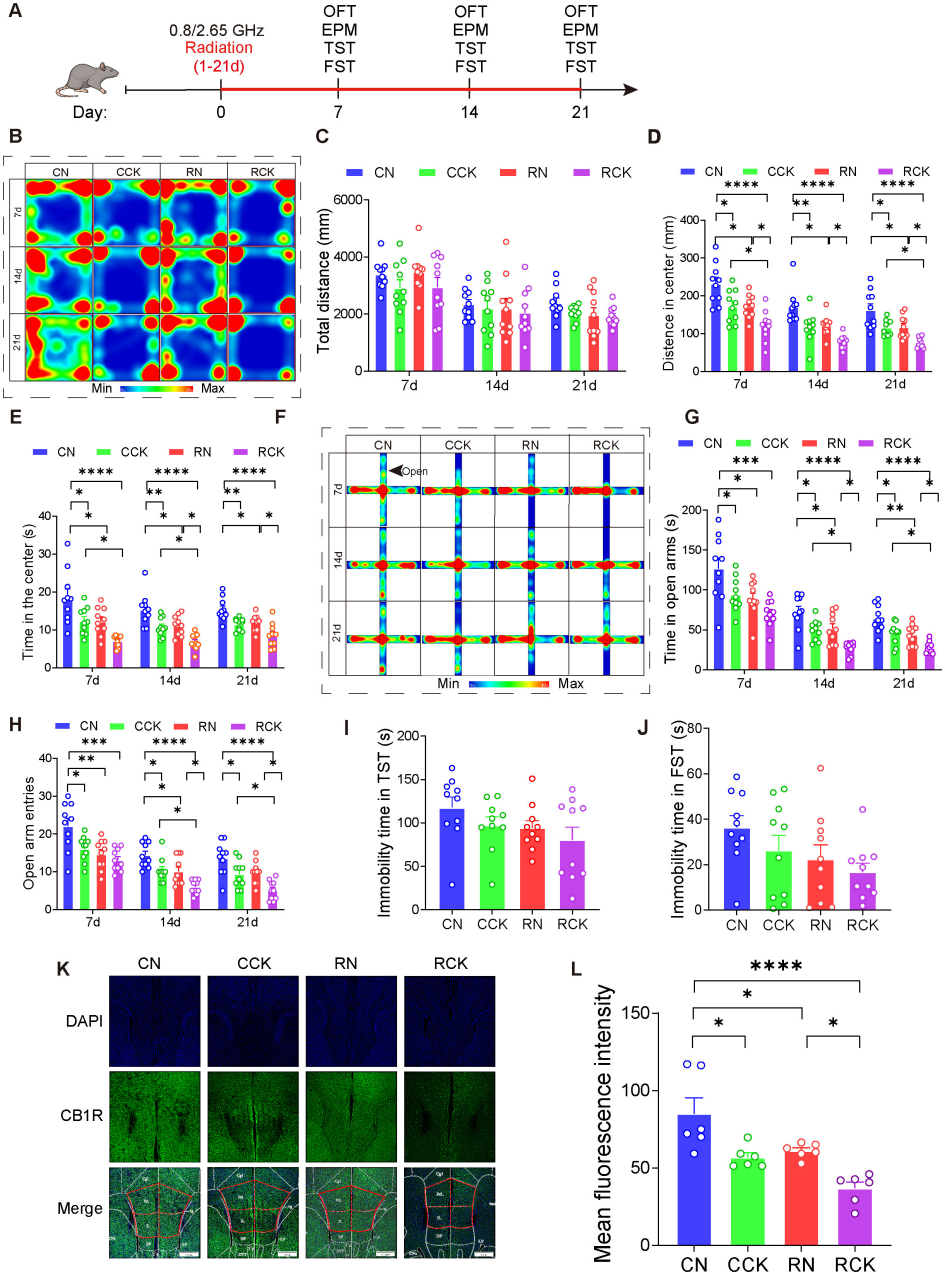
Knockdown of CB1R in the mPFC exacerbated anxiety in male mice induced by electromagnetic radiation. **(A)** Time axis of dual-frequency electromagnetic radiation (0.8/2.65 GHz, 4 W/Kg, 4h/day, 21 days) for behavioral tests (RN group: 10:00–12:00, 0.8 GHz; 12:00–14:00, 2.65 GHz; RCK group: 14:00–16:00, 0.8 GHz; 16:00–18:00, 2.65 GHz). **(B-E)** Open field test (OFT, 7d, 14d, 21d, 8:00–12:00); Representative heat map for statistical analysis of OFT results (7d, 14d, 21d, *n* = 10/group) **(B)**, total distance **(C)**, central distance **(D)**, and time spent in the center **(E)**. **(F–H)** Elevated plus maze test (EPM, 7d, 14d, 21d, 18:00–22:00); Representative heat map for statistical analysis of EPM results (7d, 14d, 21d, *n* = 10/group) **(F)**, time spent in the open arms **(G)**, and the number of open arm entries **(H)**. **(I)** Tail suspension test (TST, 20d, 18:00–22:00), relative immobility time (*n* = 10/group). **(J)** Forced swim test (FST, 22d, 8:00–12:00), relative immobility time (*n* = 10/group). **(K, L)** Immunofluorescence of CB1R in the mPFC: Representative CB1R, DAPI, and merged images **(K)**, and mean CB1R fluorescence intensity in the mPFC (*n* = 6/group) **(L)**. All data are expressed as means ± SEM, *p < 0.05, **p < 0.01, ***p < 0.001, ****p < 0.0001. All results were analyzed using the one-way ANOVA. CN, Control + NC; CCK, Control + Cnr1 knockout; RN, Radiation (0.8/2.65 GHz) + NC; RCK, Radiation (0.8/2.65 GHz) + Cnr1 knockout.

We have found that overexpressing CB1R of mPFC can reduce anxiety-like behavior in mice, so whether Knock-down CB1R of mPFC can aggravate anxiety-like behavior in mice is worth studying. After 21 days of injecting either Cnr1-knockout virus (pAAV-U6-shRNA(Cnr1)-CMV-EGFP-WPRE; 4.45E+12 v.g./ml) or NC control virus (pAAV-U6-shRNA(NC)-CMV-EGFP-WPRE; 3.10E+13 v.g./ml diluted 7 times to 4.43E+12 v.g./ml) into the mPFC, we conducted EMR modeling and behavioral tests (Figure 5A).

The results showed that, in the OFT (Figures 5B–E), compared with the RN (Radiation 0.8/2.65 GHz + NC) group, The RCK (Radiation 0.8/2.65 GHz + Cnr 1 knockout) group exhibited a significant decreased center distance on day 7 (*p* = 0.0465, Figure 5D), the center distance was significantly reduced on day 14 (*p* = 0.0377, Figure 5D), the center distance was significantly reduced on day 21 (*p* = 0.0376, Figure 5D); and the central time at 14d (*p* = 0.0137, Figure 5E) and at day 21 (*p* = 0.0261, Figure 5E) decreased in the OFT. In the EPM (Figures 5F–H), the time in open arms of RCK (radiation 0.8/2.65 GHz + Cnr 1 knockdown) was significantly less than RN (radiation 0.8/2.65 GHz + NC) on day 14 (*p* = 0.0112, Figure 5G) and on day 21 (*p* = 0.042, Figure 5G); the entries in open arms of RCK (radiation 0.8/2.65 GHz + Cnr 1 knockdown) was significantly less than RN (radiation 0.8/2.65 GHz + NC) on day 14 (*p* = 0.0271, Figure 5H) and on day 21 (*p* = 0.0194, Figure 5H). But there is no difference between RN and RCK group for TST (Figure 5I) and FST (Figure 5J). These results demonstrate that knock-down of Cnr1 in the mPFC significantly aggravated their anxiety behaviors induced by dual-frequency EMR.

Last, we detected the CB1R expression (Figures 5K–L), uncovering that the RCK group demonstrated a significant decrease (*p* = 0.0357, Figure 5L) compared to the RN group. These results signify that dual-frequency EMR induces downregulation of CB1R of mPFC, which knock-down of CB1R in the mPFC can efficiently aggravate.

### 2.6 Exposure to dual-frequency (0.8/2.65 GHz) EMR induces anxiety-like behavior in female mice and significantly decreases the content of CB1R in the medial prefrontal cortex and base lateral amygdala

**Figure 6:**
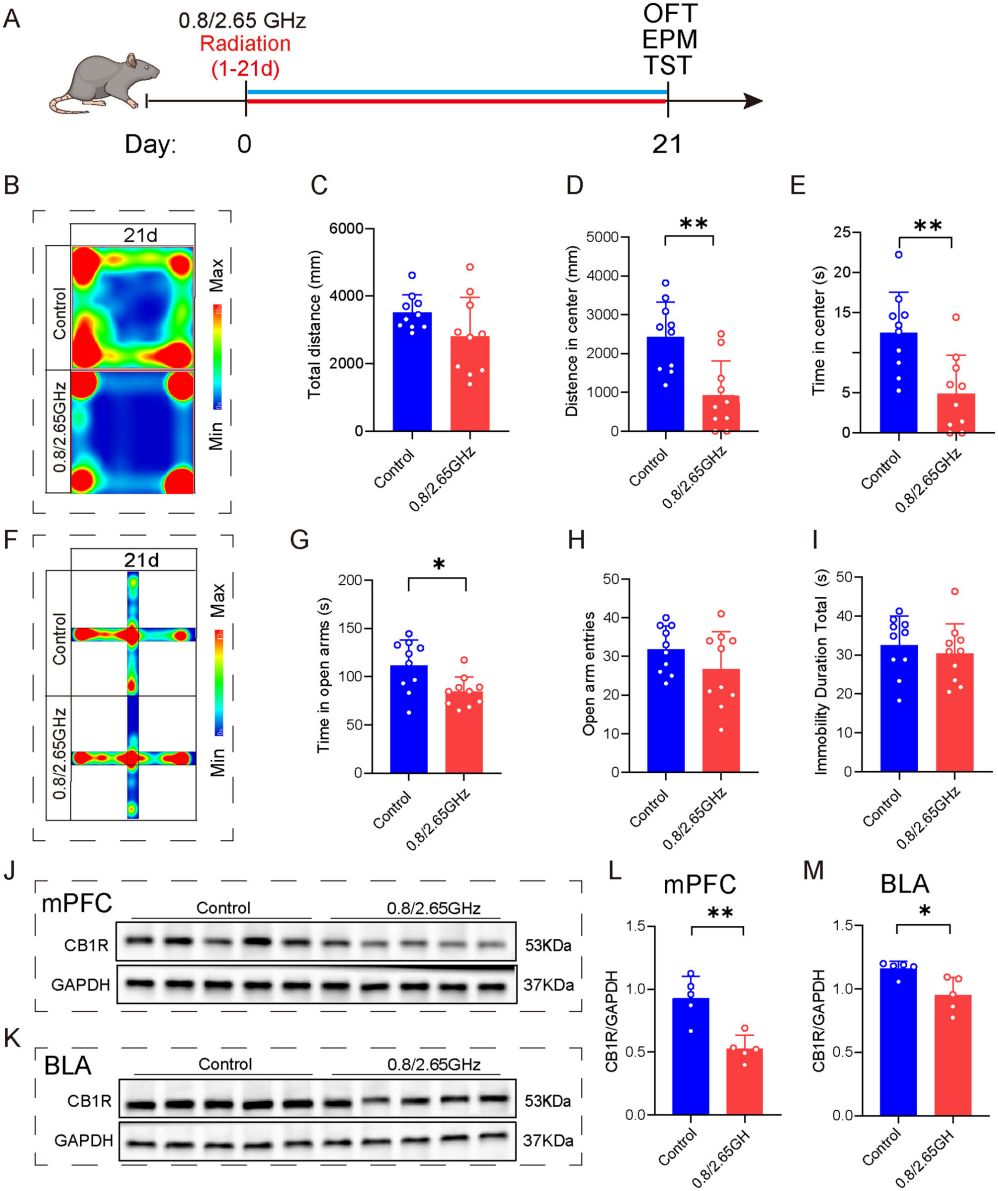
Effects of 0.8/2.65 GHz dual-frequency electromagnetic radiation on the emotions of female mice and CB1R expression in different brain regions. **(A)** Time axis of dual-frequency electromagnetic radiation (0.8/2.65 GHz, 4 W/Kg, 4h/day, 21 days) for behavioral tests (14:00–16:00, 0.8 GHz; 16:00–18:00, 2.65 GHz). **(B–E)** Open field test (OFT, 21d, 9:00–11:00); Representative heat map for statistical analysis of OFT results (21d, *n* = 10/group) **(B)**, total distance **(C)**, central distance **(D)**, and time spent in the center **(E)**. **(F–I)** Elevated plus maze test (EPM, 21d, 19:00–21:00, *n* = 10/group); Representative heat map for statistical analysis of EPM results (21d, *n* = 10/group) **(F)**, time spent in the open arms **(G)**, and the number of open arm entries **(H)**. **(I)** Tail suspension test (TST, 20d, 19:00-21:00), relative immobility time. **(J–M)** Effects of electromagnetic radiation on CB1R in the mPFC and BLA of female mice; **(J)** Western blot images showing target protein CB1R and internal control GAPDH in the mPFC, **(L)** Statistical analysis of CB1R/GAPDH protein levels in the mPFC (*n* = 5/group), **(K)** WB images showing CB1R and GAPDH in the BLA, **(M)** Statistical analysis of CB1R/GAPDH protein levels in the BLA (*n* = 5/group). All data are expressed as means ± SEM, *p < 0.05, **p < 0.01, All results were analyzed using the unpaired *t*-test.

In order to study the differences between males and females, we also conducted similar behavioral tests on female mice (Figure 6A). In the OFT (Figures 6B–E), the dual-frequency EMR group showed a significant decrease in center distance (*p* = 0.0014, Figure 6D) and center time (*p* = 0.0027, Figure 6E) compared to the control group on day 21. Similarly, in the EPM (Figures 6F–H), the dual-frequency EMR group had significantly reduced time (*p* = 0.012, Figure 6G) in the open arms on day 21. However, in comparison with the control group, the dual-frequency EMR group showed no significant difference in the total immobility duration during the TST on day 21 (Figure 6I), suggesting that dual-frequency EMR did not induce depression-like behavior in mice. These findings indicate that dual-frequency (0.8/2.65 GHz) EMR induced anxiety-like behavior in female mice.

To investigate the molecular mechanisms triggering anxiety-like behaviors, we performed WB detection of mPFC and BLA brain tissue (Figures 6J–M), found that compared to the control group, WB confirmed a significant reduction in CB1R protein expression in the mPFC (*p* = 0.0022, Figure 6L) and BLA (*p* = 0.0148, Figure 6M), indicating that prolonged exposure to dual-frequency EMR impacted the CB1R expression of the ECS within the female mice.

## 3 Discussion

This study confirms that dual-frequency EMR induces anxiety-like behavior in mice, linked to disrupted ECS function in the mPFC and BLA. fMRI showed increased ALFF in the mPFC, while molecular analysis revealed reduced CB1R levels and decreased 2-AG and AEA, leading to ECS downregulation. Overexpression of CB1R in the mPFC alleviated anxiety-like behavior, whereas CB1R knockout exacerbated it. Similar ECS dysfunction was observed in the BLA. Both male and female mice exhibited comparable responses. These findings highlight CB1R of ECS in the mPFC as a key mechanism in EMR-induced anxiety, suggesting potential therapeutic targets.

The CB1R is distributed throughout the brain and plays a neuromodulatory role in various neurotransmitter systems (Noriega-Prieto et al., 2023), with its highest concentrations found in limbic regions such as the prefrontal cortex, amygdala, and hippocampus (Burns et al., 2007). It is closely associated with stress, emotion regulation, reward processing, and fear learning (Jacob et al., 2009; Gao et al., 2024; Lucindo et al., 2025). In our previous research, we found that dual-frequency EMR leads to downregulation of CB1R in the cerebral cortex of mice (Xue et al., 2024). However, the specific brain regions where dual-frequency EMR induces anxiety remain unclear. To investigate this, we first identified a significant increase in ALFF in the mPFC of irradiated mice using fMRI, suggesting increased activity in this brain region. We then aimed to determine whether the ECS in the mPFC shows any changes. IF, QPCR, and WB experiments revealed a significant decrease in CB1R levels in the mPFC, a region known to regulate fear and anxiety expression (Adhikari et al., 2015). This indicates that the mPFC plays a crucial role in the regulation of dual-frequency EMR-induced anxiety in mice. Finally, using both overexpression and knockdown of CB1R in the mPFC, we found that CB1R overexpression alleviated anxiety-like behaviors, while CB1R knockdown exacerbated them. Thus, CB1R in the mPFC plays an essential role in regulating anxiety-like behavior induced by dual-frequency EMR in mice. Of course, there is still some room for research in AOM, CPU, Acb, S1 and other brain nuclei, which show differences in MRI.

In addition, changes in the levels of eCBs in the central nervous system can also lead to emotional changes. eCBs are synthesized “on-demand” and are released from postsynaptic neurons into the synapse, where they inhibit presynaptic activity in a retrograde manner (Herkenham et al., 1991; Marsicano and Kuner, 2008; Kendall and Yudowski, 2016). Studies have reported that eCB concentrations can be modulated by both acute and chronic stress (deRoon-Cassini et al., 2020), and disruptions in eCB signaling are associated with several stress-related disorders, including anxiety, depression, post-traumatic stress disorder (PTSD), obesity, and diabetes (Engeli, 2008; Kurtov et al., 2024; Hill et al., 2018; Charytoniuk et al., 2020; Mechoulam and Parker, 2013; Cheung et al., 2019). In this study, we found that dual-frequency EMR reduces endocannabinoids 2-AG and AEA in the mPFC, leading to a downregulation of the ECS. The activity of AEA and 2-AG is primarily regulated by synthesizing enzymes NAPE-PLD and DAGL for AEA and 2-AG, respectively or degrading enzymes FAAH and MAGL. In this study, we observed a reduction in DAGLα, DAGLβ, and NAPE-PLD, as well as an increase in FAAH and MAGL in the mPFC, consistent with the changes in endocannabinoid levels. This suggests that alterations in the levels of endocannabinoids and their enzymes in the mPFC are also involved in dual-frequency EMR-induced anxiety-like behavior in mice.

Under physiological conditions, the mPFC exerts top-down inhibition on BLA activity, preventing the expression of negative emotions (Rosenkranz and Grace, 2001; Motzkin et al., 2015; Quirk et al., 2003). However, under prolonged stress, the regulatory influence of the mPFC on the BLA is weakened, leading to overactivity in the BLA and the development of anxiety (Quirk and Gehlert, 2003; Correll et al., 2005). Anatomical evidence indicates that projection fibers from mPFC neurons primarily target the BLA (Mcdonald et al., 1996; McDonald, 1998), highlighting extensive structural and functional connectivity between the two regions, which play a crucial role in regulating fear, anxiety, and other emotions (Likhtik et al., 2014). Given the BLA's critical role in emotional regulation and its close connection with the mPFC, we also conducted molecular analyses on the BLA, finding reductions in Cnr1, CB1R, and endogenous cannabinoid 2-AG. Thus, as a core brain region involved in the regulation and expression of emotions, the BLA also participates in the anxiety-like behavior induced in mice by dual-frequency EMR exposure.

In addition, to verify sex differences, we conducted related experiments with female mice and found that dual-frequency EMR also induced anxiety in female mice. Additionally, CB1R in the mPFC and BLA was significantly reduced, further confirming the critical role of CB1R in these brain regions in dual-frequency EMR-induced negative emotions in mice.

However, our study has certain limitations. First, dual-frequency EMR may not fully replicate real-world conditions. Second, our research focused solely on the molecular mechanisms underlying the effects of dual-frequency EMR, leaving circuit-level mechanisms unexplored. Third, we studied the impact of EMR on mouse anxiety-like behavior but lacked investigations involving humans. In future research, we aim to further study the mPFC, activating or inhibiting its neurons and examining its connections with upstream and downstream brain regions to analyze the relationship between EMR-induced anxiety and neural circuits. Additionally, we plan to target CB1R in the mPFC to develop targeted interventions or treatments for alleviating EMR-induced anxiety symptoms, paving the way for potential clinical applications.

## 4 Materials and methods

### 4.1 Animals

Eight-week-old male C57BL/6J mice (20.00 ± 0.43 g) were obtained from SPF Biotechnology Co., Ltd. (Beijing, China). They were housed under standard laboratory conditions with a 12-h light-dark cycle (lights on from 7:30 to 19:30), an ambient temperature of 22 ± 2°C, and relative humidity of 50–60%. The mice were provided with unrestricted access to a standard diet and tap water. All experimental animal procedures were approved by the Institutional Animal Care and Use Committee of the National Beijing Center for Drug Safety Evaluation and Research.

### 4.2 EMR exposure equipment

The electromagnetic reverberation chamber (RC) utilized in this experiment was developed by Wu Tongning's team at the Department of Environment and Security, China Institute of Information and Communication Technology (Li et al., 2016). Constructed with reinforced concrete, the RC is a large shielded enclosure featuring highly conductive reflective walls and multiple mechanical stirrers. The stirrers' rotation alters the chamber's boundary conditions, creating a statistically uniform, isotropic, and randomly polarized electromagnetic environment. Key components of the RC include signal generators, power amplifiers, and shielding structures. The chamber can produce electromagnetic waves within a frequency range of 0 to 3 GHz. In this study, 0.8 and 2.65 GHz frequencies were applied at a dose of 4 W/kg. The electric field intensity was calculated based on the mice's average body weight, with specific experimental parameters detailed in Table 1.

**Table 1:** Corresponding electric field strength parameters in mice with different body weights and frequencies.

**Weight of mice (g)**	**Radiation frequency (GHz)**	**Electric field intensity (V/m)**
21.9	2.65	188.93
25.0	2.65	189.79
30.0	2.65	190.00
21.9	0.80	243.00
25.0	0.80	262.00
30.0	0.80	285.00

### 4.3 OFT

The open field test (OFT) apparatus, obtained from Shanghai Xinruan Technology Co., Ltd. (Shanghai, China), consists of an open polypropylene box with dimensions of 50 × 50 × 40 cm3. During the test, each mouse is placed in a designated corner of the box and allowed to explore freely. Spontaneous activity is recorded and analyzed using specialized software over a 5-min session. Key parameters, including total distance traveled, distance covered in the center, and time spent in the center, are used to evaluate anxiety-like behaviors. Prior to testing, mice are acclimated to the experimental environment for at least 2 h. The apparatus is thoroughly cleaned with a 75% ethanol solution between trials to prevent olfactory cues from influencing results.

### 4.4 EPM

The apparatus, acquired from Anhui Zhenghua Biological Instrument Equipment Co., Ltd. (Anhui, China), stands 50 cm tall and features two open arms and two closed arms (50 cm long, 5 cm wide, with 15 cm high walls). A camera records the mouse's movements for 5 min, with behavior analyzed using specialized software. Key parameters, such as distance traveled in the open arms, time spent in the open arms, and the number of open-arm entries, are used to evaluate anxiety-like behaviors in mice. To minimize stress, mice are acclimated to the testing environment for at least 2 h before the experiment. A quiet environment is maintained throughout the procedure. The maze is cleaned with 75% ethanol between trials to eliminate any residual odors that could affect the results.

### 4.5 TST

The apparatus, obtained from Anhui Zhenghua Biological Instrument Equipment Co., Ltd. (Anhui, China), was used for the experiment. Before beginning, a rubber band was secured around one-third of the mouse's tail and fastened with a clip, suspending the mouse in an inverted position 30 cm above the ground within an observation box of a behavioral analysis system. Video equipment was activated to automatically record the mouse's activity for 6 min, with the first 2 min serving as an adaptation phase and the remaining 4 min as the test phase. Animal behavior software analyzed the relative immobility time during the 4-min test phase to evaluate depression-like behavior. A quiet environment was maintained throughout the experiment, and the apparatus was cleaned with alcohol after each trial to ensure hygiene and eliminate potential interferences.

### 4.6 EZM

The apparatus, manufactured by Shanghai Xinruan Technology Co., Ltd. (Shanghai, China), is an elevated zero maze (EZM) constructed from dark gray aluminum. It features a circular platform with a 5 cm wide “O”-shaped corridor elevated 50 cm above the ground. The maze has an inner diameter of 55 cm and an outer diameter of 60 cm, divided into four quadrants: two opposing enclosed quadrants and two opposing open quadrants. The open quadrants lack side walls, while the enclosed quadrants are surrounded by 15 cm high side walls. At the start of the experiment, the mouse is placed at the midpoint of an open quadrant, facing a closed quadrant, and observed for 5 min. Anxiety-like behavior is evaluated using two parameters: the time spent in the enclosed quadrants and the total number of entries into the open quadrants. A quiet environment is maintained during testing, and the apparatus is cleaned with alcohol after each trial to ensure consistency and hygiene.

### 4.7 SIT

The apparatus, manufactured by Shanghai Xinruan Technology Co., Ltd. (Shanghai, China), includes A cylindrical cage with a diameter of 10 cm positioned along one side of an open field (50 cm × 50 cm × 50 cm). A C57BL/6J mouse is initially placed in a corner of the open field, and its movement trajectory is recorded and analyzed for 2.5 min using a camera. Then, a novel CD-1 mouse is introduced into a transparent plastic cage, and a new 2.5-min monitoring and recording session is conducted. The open field is cleaned with 75% ethanol before testing the next C57BL/6J mouse. The social interaction zone, measuring 20 cm × 15 cm, is used to record the time the mouse spends in this zone, referred to as “social time.” The social interaction ratio (SIR), an indicator of social anxiety, is calculated by dividing the time spent in the interaction zone in the presence of the CD-1 mouse by the time spent in the absence of the CD-1 mouse. A quiet environment is maintained during the experiment, and the apparatus is thoroughly cleaned with alcohol between trials.

### 4.8 LDBT

The light-dark box, obtained from Shanghai Xinruan Software Technology Co., Ltd. (Shanghai, China), is a rectangular apparatus measuring 42 × 21 × 25 cm. It is divided into a small dark compartment (occupying one-third of the box) and a larger illuminated compartment (occupying two-thirds of the box). A small opening, 3 cm high and 4 cm wide, connects the two compartments. The light intensity in the illuminated compartment ranges from 200 to 400 lux or higher, while the dark compartment maintains an intensity of 5 lux or lower. During the experiment, the mouse is allowed to move freely between the two compartments for 5 min. Specialized software records the latency time for the mouse to enter the illuminated compartment, a parameter used to evaluate anxiety-like behavior. A quiet environment is maintained throughout the experiment, and the apparatus is cleaned with alcohol after each trial to ensure consistency and hygiene.

### 4.9 FST

The apparatus, purchased from Anhui Zhenghua Biological Instrument Equipment Co., Ltd. (Anhui, China), consists of a forced swim cylinder for mice with a diameter of 10 cm and a height of 25 cm. Before the experiment, the cylinder is filled with warm water maintained at a temperature of 23 to 25°C. The water level is set to approximately 15 cm to ensure the mouse's tail does not touch the bottom. Once the mouse is placed in the water, recording begins using specialized software for a total duration of 6 min, with the first 2 min serving as an adaptation phase. The relative immobility time during the final 4 minutes is analyzed to evaluate depression-like behavior in the mice. Prior to the experiment, the mouse is acclimated to the testing environment for at least 2 h. A quiet environment is maintained throughout the experiment, and fresh water is used for each trial to ensure consistency and hygiene.

### 4.10 Functional magnetic resonance imaging

Using a small animal imaging system (PharmaScan 70/16US, Bruker, Germany), both control group mice and dual-frequency EMR group mice were pre-anesthetized with a mixture of 2.5–3.0% isoflurane and oxygen. The mice were positioned prone on the animal bed, and their respiratory rate was continuously monitored in real-time using a physiological state monitoring system (PC-SAM32 software, Small Animal Instruments Inc., Stony Brook, NY). The anesthetic dosage was adjusted as needed to maintain a respiratory rate between 61 and 80 breaths per minute. To keep the mice warm during imaging, a water bath (SC100-S14P, Thermo Scientific) was used. Resting-state functional data were collected using Paravision 6.0.1 software. First, a localizing scan was performed to confirm proper positioning of the mice, using scanning parameters set to TR/TE = 100/3 ms, 1 slice, 1 mm slice thickness, image size 256 × 256, and a field of view of 40 × 40 mm. Next, brain structural images were acquired using the T2_TurboRare sequence with parameters: TR/TE = 4,500/35 ms, 40 slices, 3 averages, 0.35 mm slice thickness, image size 256 × 256, and a field of view of 20 × 20 mm. Finally, resting-state functional images were collected using a GRE sequence (T2star_FID_EPI_sat) with the following parameters: TR/TE = 2,000/15 ms, 40 slices, 1 average, 300 repetitions, 0.35 mm slice thickness, image size 64 × 64, field of view 20 × 20 mm, and bandwidth 2,00,000 Hz. Subsequent analysis of ALFF and ReHo in both control and radiation group mice will be conducted by Shanghai Ji Ying Technology Co., Ltd.

### 4.11 Serum hormone test

Following the completion of behavioral experiments, the mice were anesthetized via intraperitoneal injection of 1% sodium pentobarbital. Once anesthesia was confirmed, blood samples were collected through cardiac puncture. The collected blood was stored at 4°C overnight and subsequently centrifuged at 4,000 rpm for 10 min to isolate the serum. Serum levels of CORT and CRH were determined using a mouse CORT/CRH ELISA kit (Sankang Biotechnology Co., Ltd.), following the manufacturer's protocol.

### 4.12 Endocannabinoid test

After anesthetizing the mice with an intraperitoneal injection of 1% sodium pentobarbital, their brains were carefully removed. The extracted brains were embedded in a freezing medium and maintained at −20°C for 30 min. Using a cryostat, sections were prepared to target the mPFC and BLA regions, which were subsequently collected with a tissue micro-sampler and stored at −80°C. Endogenous cannabinoids 2-AG and AEA were measured using pooled tissue samples from two mice per group, employing a mouse endogenous cannabinoid 2-AG/AEA ELISA kit (Shanghai Fanta Biotechnology Co., Ltd., China) in accordance with the provided protocol.

### 4.13 Western blot

The mice were anesthetized with 0.7% sodium pentobarbital before neck breakage. The brains were quickly extracted from the euthanized mice and frozen in a freezing microtome. After the brains were hard frozen, they were cut into mPFC and BLA using a freezing microtome and dug into mPFC and BLA with a microdrill sampler. The brain tissue was lysed thoroughly using an ultrasonic disruptor, followed by centrifugation to collect the supernatant. The total protein concentration was determined using a BCA kit (Thermo Fisher Scientific, USA). Protein samples were separated via 10% SDS-PAGE and transferred onto a PVDF membrane. After washing with TBST, the membrane was blocked with 5% skim milk for 1 h, then incubated overnight at 4°C with primary antibody at a 1:1,000 dilution ratio (internal controls: GAPDH, α-tubulin, β-actin, Solarbio China; primary antibodies: CB1R, Abcam, USA; FAAH, MGL, DAGLα, DAGLβ, NAPE-PLD, ImmunoWay, China). After washing with TBST, the membrane was incubated with secondary antibody (Only CB1R was goat anti-rabbit, and the remaining proteins were goat anti-rat) at a 1:2,000 dilution for 1 h to enhance specificity, followed by three additional TBST washes. Supper ECL Western Blotting Substrate from Shanghai Zeha Biotechnology Co., Ltd was applied to the membrane and the signal was captured using a gel imaging system (Bio-Rad Laboratories Co., Ltd.). The grayscale values of the Western blot bands were analyzed and quantified using ImageJ-win64 1.51 software.

### 4.14 Immunofluorescence

After anesthetizing the mice with isoflurane, their limbs were secured, and the sternum was cut open to expose the heart. A sufficient volume of physiological saline or PBS was slowly infused into the left ventricle for cardiac perfusion until the liver appeared grayish-white. At this point, the solution was replaced with pre-cooled 4% paraformaldehyde (PFA) at 4°C. Perfusion was continued until the mice's limbs became rigid. The mice were then decapitated, and the brains were carefully removed. The intact brain tissues were fixed overnight in 4% PFA and subsequently dehydrated in PBS containing 20% sucrose until the tissues sank to the bottom of the tube. The tissues were further dehydrated in PBS with 30% sucrose for 24 h. Next, the brains were sectioned into slices 30 μm thick using a cryostat. The collected brain sections were rinsed three times with PBS and immersed in a blocking solution composed of PBS with 0.2% Triton X-100, 5% goat serum, and 2.5% BSA for 2 h at room temperature. The sections were then incubated overnight at 4°C on a shaker with the primary antibody targeting CB1R (Abcam, USA; 1:1,000 dilution). The following day, the brain sections were incubated at room temperature for 1 hour and rinsed three times with PBS, each rinse lasting 10 min. After rinsing, the sections were incubated with the appropriate fluorescent secondary antibody (Goat Anti-Rabbit lgG; Abcam, USA; 1:1,000 dilution) for 2 h at room temperature. The secondary antibody was then discarded, and the sections were rinsed three additional times in PBS, with each rinse lasting 10 min. Finally, the sections were mounted onto slides using a mounting medium containing 0.1% DAPI. Using the mouse brain atlas, the medial prefrontal cortex (mPFC) and basolateral amygdala (BLA) regions were identified. Fluorescence signals were observed and photographed using a Nikon A1R laser scanning confocal microscope. The average fluorescence intensity of CB1R in the target brain regions was quantified using Image J software.

### 4.15 RT-qPCR

Immediately after decapitation, the mPFC and BLA brain tissues of the mouse were extracted using a micro tissue sampler in a cryostat. The tissues were then rapidly frozen in liquid nitrogen and stored at −80°C. RNA was extracted using TriZol reagent (Thermo Fisher, Waltham, MA, USA) and quantified with an ultramicro UV spectrophotometer (Quawell Q3000). Reverse transcription to cDNA was performed using the T100™ Thermal Cycler (Bio-Rad). Quantitative real-time PCR (qRT-PCR) was carried out using 1 μg of cDNA as the template. Amplification was performed with the StepOnePlus™ Real-Time PCR System (Thermo Fisher). β-actin was used as the reference gene, and cnr1 was the target gene. The mRNA levels were calculated using the 2-ΔCt method.

### 4.16 Stereotaxic surgery

Mice were anesthetized with 5% isoflurane and then secured in a stereotaxic apparatus (Kopf Instruments, Tujunga, CA), with anesthesia maintained at 2% isoflurane. Ocular ointment was applied, and a longitudinal incision was made on the skin at the top of the mouse's head. The exposed area was cleaned first with 3% hydrogen peroxide and then with saline to reveal the skull. The bregma's height difference was adjusted to be within ±0.05 mm, with the anterior bregma positioned 0.00–0.05 mm lower than the lambda, ensuring proper leveling. Holes were drilled at the specified coordinates on the skull, and a 10 μL microsyringe (Hamilton Co., Reno, NV) controlled by a Micropump (World Precision Instruments, Sarasota, FL) was used to inject the virus (Produced by OBiO Technology (Shanghai) Corp., Ltd) bilaterally into the mPFC. The injection rate was maintained at 30 nL/min, delivering 500 nL per side (coordinates: AP: +1.95 mm, ML: ±0.25 mm, DV: −3.00 mm). After the injection, the needle was left in place for 10 min to allow for proper diffusion. The skin was then sutured, and the incision area was cleaned and disinfected. The health of the mice was monitored post-surgery. Following a 21-day period to allow for virus expression, subsequent experiments were performed.

### 4.17 Statistical analysis

Data analysis was conducted using GraphPad Prism 8.4.2 software. A normality test was performed on the dataset prior to statistical analysis. For comparisons between two samples, an unpaired *t*-test was used, while one-way analysis of variance (ANOVA) was employed for comparisons among multiple groups. Results are presented as mean ± standard error of the mean (M ± SEM). Statistical significance was defined as follows: ^*^p < 0.05, ^**^p < 0.01, ^***^p < 0.001, ^****^p < 0.0001, with “ns” indicating no statistical significance.

## 5 Conclusion

**Figure 7:**
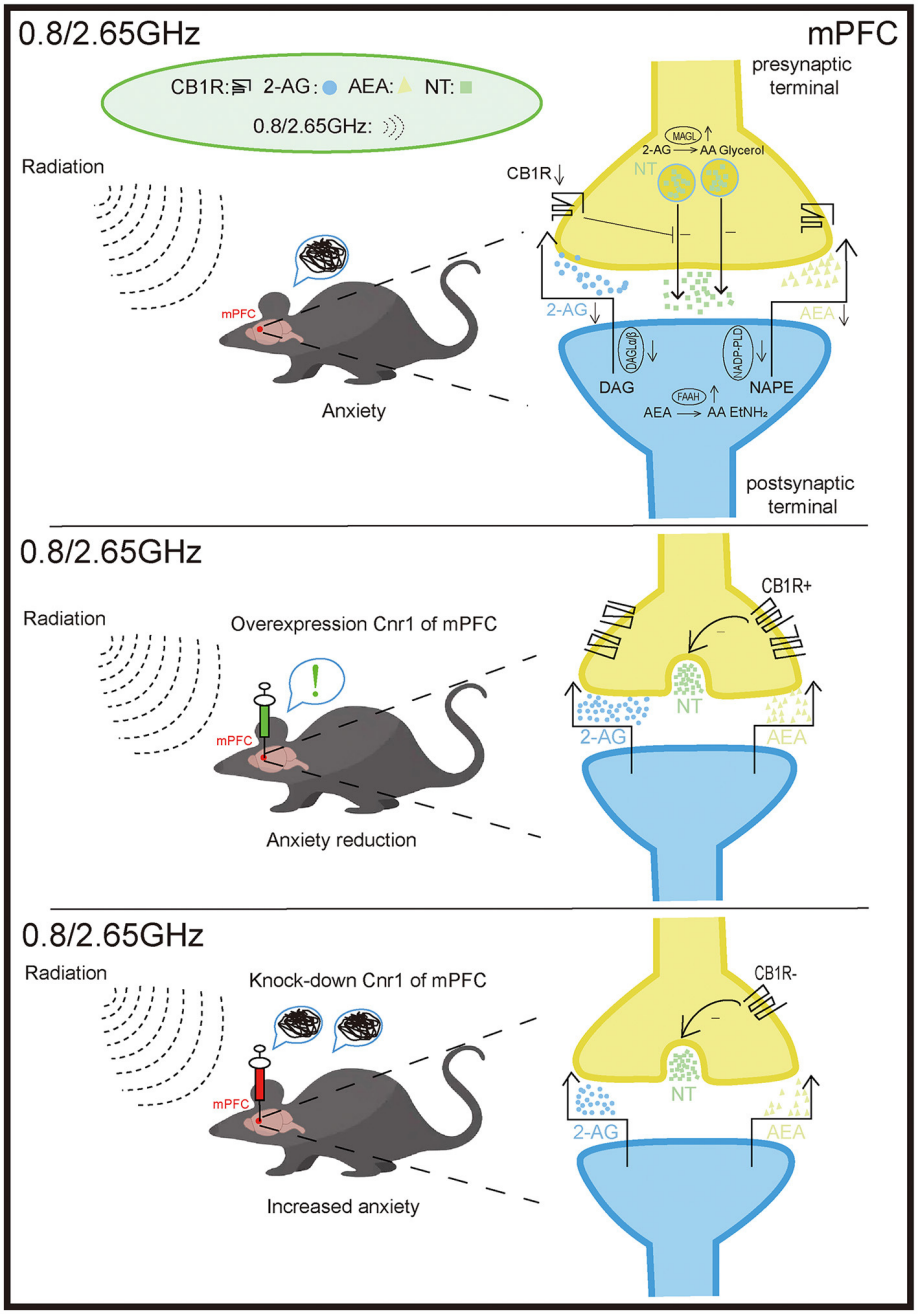
Effects of 0.8/2.65 GHz dual-frequency electromagnetic radiation (EMR) on the endocannabinoid system (ECS) in the mPFC. The anxiety-like behavior observed in mice exposed to dual-frequency EMR (0.8/2.65 GHz) is closely associated with disruptions in the ECS of the medial prefrontal cortex (mPFC). These disruptions include the downregulation of the receptor cannabinoid receptor type 1 (CB1R) and the ligands 2-arachidonoylglycerol (2-AG) and anandamide (AEA). Furthermore, Overexpression of CB1R in the mPFC significantly alleviates anxiety-like behavior in mice. In contrast, knockdown of CB1R in the mPFC exacerbates negative emotional responses. mPFC: medial prefrontal cortex. CB1R: cannabinoid receptor type 1. 2-AG, 2-arachidonoylglycerol; AEA, anandamide; DAGL-α/β, diacylglycerol lipase-α/β (synthesizes 2-AG); NAPE-PLD, N-acylphosphatidylethanolamine phospholipase D (synthesizes AEA); MAGL, monoacylglycerol lipase (degrades 2-AG); FAAH, fatty acid amide hydrolase (degrades AEA).

In summary, this study demonstrates that anxiety-like behavior induced by dual-frequency EMR is closely associated with the ECS in the mPFC and BLA. Moreover, overexpression of CB1R in the mPFC significantly alleviates anxiety-like behavior in mice, while knockdown of CB1R in the mPFC exacerbates negative emotional responses (Figure 7). This research offers new insights into potential strategies for the treatment or prevention of the effects of dual-frequency EMR.

## Data Availability

The original contributions presented in the study are included in the article/supplementary material, further inquiries can be directed to the corresponding authors.

## References

[B1] Adhikari A. Lerner T. N. Finkelstein J. Pak S. Jennings J. H. Davidson T. J.. (2015). Basomedial amygdala mediates top-down control of anxiety and fear. Nature 527, 179–185. 10.1038/nature15698 26536109 PMC4780260

[B2] Burns H. D. Van Laere K. Sanabria-Bohórquez S. Hamill T. G. Bormans G. Eng W.. (2007). [18F]MK-9470, a positron emission tomography (PET) tracer for *in vivo* human PET brain imaging of the cannabinoid-1 receptor. Proc. Natl. Acad. Sci. USA. 104, 9800–9805. 10.1073/pnas.0703472104 17535893 PMC1877985

[B3] Charytoniuk T. Zywno H. Konstantynowicz-Nowicka K. Berk K. Bzdega W. Chabowski A. (2020). Can physical activity support the endocannabinoid system in the preventive and therapeutic approach to neurological disorders? Int. J. Mol. Sci. 21:4221. 10.3390/ijms21124221 32545780 PMC7352563

[B4] Cheung K. A. K. Peiris H. Wallace G. Holland O. J. Mitchell M. D. (2019). The Interplay between the endocannabinoid system, epilepsy and cannabinoids. Int. J. Mol. Sci. 20:6079. 10.3390/ijms20236079 31810321 PMC6929011

[B5] Correll C. M. Rosenkranz J. A. Grace A. A. (2005). Chronic cold stress alters prefrontal cortical modulation of amygdala neuronal activity in rats. Biol. Psychiatry 58, 382–391. 10.1016/j.biopsych.2005.04.009 16023619

[B6] Demaili A. Portugalov A. Maroun M. Akirav I. Braun K. Bock J. (2024). Early life stress induces decreased expression of CB1R and FAAH and epigenetic changes in the medial prefrontal cortex of male rats. Front. Cell Neurosci. 18:1474992. 10.3389/fncel.2024.1474992 39503008 PMC11534599

[B7] deRoon-Cassini T. A. Stollenwerk T. M. Beatka M. Hillard C. J. (2020). Meet your stress management professionals: the endocannabinoids. Trends Mol. Med. 26, 953–968. 10.1016/j.molmed.2020.07.002 32868170 PMC7530069

[B8] Engeli S. (2008). Dysregulation of the endocannabinoid system in obesity. J. Neuroendocrinol. 20(Suppl 1), 110–115. 10.1111/j.1365-2826.2008.01683.x 18426509

[B9] Feng J. Wang X. Pan M. Li C.-X. Zhang Z. Sun M.. (2024). The medial prefrontal cortex-basolateral amygdala circuit mediates anxiety in shank3 insg3680 knock-in mice. Neurosci. Bull. 41, 77–92. 10.1007/s12264-024-01280-5 39207622 PMC11748662

[B10] Gao J. Liu Y. Xu H. Wu K. Zhang L. Cheng P.. (2024). Divergent input patterns to the central lateral amygdala play a duet in fear memory formation. iScience 27:110886. 10.1016/j.isci.2024.110886 39319272 PMC11421289

[B11] Glaser Z. Brown P. F. (1976). Bibliography of Reported Biological Phenomena (Effects) and Clinical Manifestations Attributed to Microwave and Radio-Frequency Radiation. Supplement Number 8. Available online at: https://www.semanticscholar.org/paper/Bibliography-of-Reported-Biological-Phenomena-and-Glaser-Brown/45d7955f9892849ffd3ebd1fa5130ae777d0a83a (accessed November 9, 2024).

[B12] Gunduz-Cinar O. Castillo L. I. Xia M. Van Leer E. Brockway E. T. Pollack G. A.. (2023). A cortico-amygdala neural substrate for endocannabinoid modulation of fear extinction. Neuron 111, 3053–3067.e10. 10.1016/j.neuron.2023.06.023 37480845 PMC10592324

[B13] Herkenham M. Lynn A. B. Johnson M. R. Melvin L. S. de Costa B. R. Rice K. C. (1991). Characterization and localization of cannabinoid receptors in rat brain: a quantitative in vitro autoradiographic study. J. Neurosci. 11, 563–583. 10.1523/JNEUROSCI.11-02-00563.1991 1992016 PMC6575215

[B14] Hill M. N. Campolongo P. Yehuda R. Patel S. (2018). Integrating endocannabinoid signaling and cannabinoids into the biology and treatment of posttraumatic stress disorder. Neuropsychopharmacology 43, 80–102. 10.1038/npp.2017.162 28745306 PMC5719095

[B15] Imperatore R. Morello G. Luongo L. Taschler U. Romano R. De Gregorio D.. (2015). Genetic deletion of monoacylglycerol lipase leads to impaired cannabinoid receptor CB1R signaling and anxiety-like behavior. J. Neurochem. 135, 799–813. 10.1111/jnc.13267 26223500

[B16] Jacob W. Yassouridis A. Marsicano G. Monory K. Lutz B. Wotjak C. T. (2009). Endocannabinoids render exploratory behaviour largely independent of the test aversiveness: role of glutamatergic transmission. Genes, Brain and Behavior 8, 685–698. 10.1111/j.1601-183X.2009.00512.x 19563475

[B17] Kendall D. A. Yudowski G. A. (2016). Cannabinoid receptors in the central nervous system: their signaling and roles in disease. Front. Cell Neurosci. 10:294. 10.3389/fncel.2016.00294 28101004 PMC5209363

[B18] Kondev V. Najeed M. Yasmin F. Morgan A. Loomba N. Johnson K.. (2023). Endocannabinoid release at ventral hippocampal-amygdala synapses regulates stress-induced behavioral adaptation. Cell Rep. 42:113027. 10.1016/j.celrep.2023.113027 37703881 PMC10846613

[B19] Kurtov M. Rubini,ć I. Liki,ć R. (2024). The endocannabinoid system in appetite regulation and treatment of obesity. Pharmacol. Res. Perspect. 12:e70009. 10.1002/prp2.70009 39292202 PMC11409765

[B20] Li C. Yang L. Lu B. Xie Y. Wu T. A. (2016). A reverberation chamber for rodents' exposure to wideband radiofrequency electromagnetic fields with different small-scale fading distributions. Electromagn. Biol. Med. 35, 30–39. 10.3109/15368378.2014.960086 25259622

[B21] Li Y. (2024). Effect of Xiaoyaosan on brain volume and microstructure diffusion changes to exert antidepressant-like effects in mice with chronic social defeat stress. Front. Psychiatry 15:1414295. 10.3389/fpsyt.2024.1414295 39371910 PMC11450227

[B22] Likhtik E. Stujenske J. M. Topiwala M. A. Harris A. Z. Gordon J. A. (2014). Prefrontal entrainment of amygdala activity signals safety in learned fear and innate anxiety. Nat. Neurosci. 17, 106–113. 10.1038/nn.3582 24241397 PMC4035371

[B23] Lu H. C. Mackie K. (2015). An introduction to the endogenous cannabinoid system. Biol. Psychiatry 79, 516–525. 10.1016/j.biopsych.2015.07.028 26698193 PMC4789136

[B24] Lucindo M. S. S. Albuquerque A. L. S. Pereira K. A. Salgado K. D. C. B. Oliveira L. A. M. Engel D. F.. (2025). Chronic cannabidiol administration modulates depressive and cognitive alterations induced by social isolation in male mice. Behav. Brain Res. 480:115408. 10.1016/j.bbr.2024.115408 39725273

[B25] Lutz B. Marsicano G. Maldonado R. Hillard C. J. (2015). The endocannabinoid system in guarding against fear, anxiety and stress. Nat. Rev. Neurosci. 16:705. 10.1038/nrn4036 26585799 PMC5871913

[B26] Marcus D. J. Bedse G. Gaulden A. D. Ryan J. D. Kondev V. Winters N. D.. (2020). Endocannabinoid signaling collapse mediates stress-induced amygdalo-cortical strengthening. Neuron 105, 1062–1076.e6. 10.1016/j.neuron.2019.12.024 31948734 PMC7992313

[B27] Marsicano G. Kuner R. (2008). “Anatomical distribution of receptors, ligands and enzymes in the brain and in the spinal cord: circuitries and neurochemistry,” in Cannabinoids and the Brain, ed. A. Köfalvi (Boston, MA: Springer). 10.1007/978-0-387-74349-3_10

[B28] McDonald A. J. (1998). Cortical pathways to the mammalian amygdala. Prog. Neurobiol. 55, 257–332. 10.1016/S0301-0082(98)00003-3 9643556

[B29] Mcdonald A. J. Mascagni F. Guo L. (1996). Projections of the medial and lateral prefrontal cortices to the amygdala: a Phaseolus vulgaris leucoagglutinin study in the rat. Neuroscience 71, 55–75. 10.1016/0306-4522(95)00417-3 8834392

[B30] Mechoulam R. Parker L. A. (2013). The endocannabinoid system and the brain. Annu. Rev. Psychol. 64, 21–47. 10.1146/annurev-psych-113011-143739 22804774

[B31] Morena M. Leitl K. D. Vecchiarelli H. A. Gray J. M. Campolongo P. Hill M. N. (2016). Emotional arousal state influences the ability of amygdalar endocannabinoid signaling to modulate anxiety. Neuropharmacology 111, 59–69. 10.1016/j.neuropharm.2016.08.020 27553121

[B32] Motzkin J. C. Philippi C. L. Wolf R. C. Baskaya M. K. Koenigs M. (2015). Ventromedial prefrontal cortex is critical for the regulation of amygdala activity in humans. Biol. Psychiatry 77, 276–284. 10.1016/j.biopsych.2014.02.014 24673881 PMC4145052

[B33] Noriega-Prieto J. A. Kofuji P. Araque A. (2023). Endocannabinoid signaling in synaptic function. Glia 71, 36–43. 10.1002/glia.24256 36408881 PMC9679333

[B34] Quirk G. J. Gehlert D. R. (2003). Inhibition of the amygdala: key to pathological states? Ann. N. Y. Acad. Sci. 985, 263–272. 10.1111/j.1749-6632.2003.tb07087.x 12724164

[B35] Quirk G. J. Likhtik E. Pelletier J. G. Par,é D. (2003). Stimulation of medial prefrontal cortex decreases the responsiveness of central amygdala output neurons. J. Neurosci. 23, 8800–8807. 10.1523/JNEUROSCI.23-25-08800.2003 14507980 PMC6740415

[B36] Raines J. K. (1981). Electromagnetic field interactions with the human body: observed effects and theories - NASA Technical Reports Server (NTRS). Available online at: https://ntrs.nasa.gov/citations/19810017132 (accessed November 9, 2024).

[B37] Rohrlich F. (1961). The definition of electromagnetic radiation. IL Nuovo Cimento 21, 811–822. 10.1007/BF02785607

[B38] Rosenkranz J. A. Grace A. A. (2001). Dopamine attenuates prefrontal cortical suppression of sensory inputs to the basolateral amygdala of rats. J. Neurosci. 21, 4090–4103. 10.1523/JNEUROSCI.21-11-04090.2001 11356897 PMC6762693

[B39] Wolf D. Oettl L.-L. Winkelmeier L. Linster C. Kelsch W. (2024). Anterior olfactory cortices differentially transform bottom-up odor signals to produce inverse top-down outputs. J. Neurosci. 44:e0231242024. 10.1523/JNEUROSCI.0231-24.2024 39266300 PMC11529817

[B40] Wu Y. Lu L. Qing T. Shi S. Fang G. (2024). Transient increases in neural oscillations and motor deficits in a mouse model of parkinson's disease. IJMS 25:9545. 10.3390/ijms25179545 39273491 PMC11394686

[B41] Xue T. Ma R.-H. Xu C. Sun B. Yan D.-F. Liu X.-M.. (2024). The endocannabinoid system is involved in the anxiety-like behavior induced by dual-frequency 2.65/0.8 GHz electromagnetic radiation in mice. Front. Mol. Neurosci. 17:1366855. 10.3389/fnmol.2024.1366855 38685914 PMC11057378

[B42] Yue L. Bao C. Zhang L. Zhang F. Zhou W. Iannetti G. D.. (2025). Neuronal mechanisms of nociceptive-evoked gamma-band oscillations in rodents. Neuron 113, 1–16. 10.1016/j.neuron.2024.12.011 39809278

[B43] Zheng R. Zhang X. Gao Y. Gao D. Gong W. Zhang C.. (2023). Biological effects of exposure to 2650 MHz electromagnetic radiation on the behavior, learning, and memory of mice. Brain and Behavior 13:e3004. 10.1002/brb3.3004 37118929 PMC10275548

[B44] Zou S. Kumar U. (2018). Cannabinoid receptors and the endocannabinoid system: signaling and function in the central nervous system. Int. J. Mol. Sci. 19:833. 10.3390/ijms19030833 29533978 PMC5877694

